# Coding and non-coding variants in the ciliopathy gene *CFAP410* cause early-onset non-syndromic retinal degeneration

**DOI:** 10.21203/rs.3.rs-3871956/v1

**Published:** 2024-02-09

**Authors:** Riccardo Sangermano, Priya Gupta, Cherrell Price, Jinu Han, Julien Navarro, Christel Condroyer, Emily M. Place, Aline Antonio, Shizuo Mukai, Xavier Zanlonghi, José-Alain Sahel, Jacque L. Duncan, Eric A. Pierce, Christina Zeitz, Isabelle Audo, Rachel M. Huckfeldt, Kinga M. Bujakowska

**Affiliations:** 1 Ocular Genomics Institute, Massachusetts Eye and Ear Infirmary, Department of Ophthalmology, Harvard Medical School, Boston, MA, USA; 2 Institute of Vision Research, Department of Ophthalmology, Gangnam Severance Hospital, Yonsei University College of Medicine, Seoul, Republic of Korea; 3 Sorbonne Université, INSERM, CNRS, Institut de la Vision, Paris, France; 4 Retina Service, Massachusetts Eye and Ear, Department of Ophthalmology, Harvard Medical School, Boston, MA, USA; 5 Centre de compétence maladies rares, Service d’Ophtalmologie, CHU Rennes, Rennes, France; 6 Centre Hospitalier National d’Ophtalmologie des Quinze-Vingts, Centre de Référence Maladies Rares REFERET and INSERM-DGOS CIC 1423, Paris, France; 7 Vision Institute, University of Pittsburgh Medical Center and School of Medicine, Pennsylvania, USA; 8 Department of Ophthalmology, University of California, San Francisco, California, USA.

## Abstract

Inherited retinal degenerations are blinding genetic disorders characterized by high genetic and phenotypic heterogeneity. The implementation of next-generation sequencing in routine diagnostics, together with advanced clinical phenotyping including multimodal retinal imaging, have contributed to the increase of reports describing novel genotype-phenotype associations and phenotypic expansions. In this study, we describe sixteen families with early-onset non-syndromic retinal degenerations in which affected probands carried rare bi-allelic variants in *CFAP410*, a ciliary gene previously associated with syndromic recessive Jeune syndrome. The most common retinal phenotypes were cone-rod and rod-cone dystrophies, but the clinical presentations were unified by their early onset as well as the severe impact on central visual function. Twelve variants were detected (three pathogenic, seven likely pathogenic, two of uncertain significance), eight of which were novel. One deep intronic change, c.373+91A>G, led to the creation of a cryptic splice acceptor site in intron four, followed by the inclusion of a 200- base pair pseudoexon and subsequent premature stop codon formation. To our knowledge this is the first likely pathogenic deep-intronic variant identified in this gene. Meta-analysis of all published and novel *CFAP410* variants revealed no clear correlation between the severity of the *CFAP410-*associated phenotypes and the identified causal variants. This is supported by the fact that the frequently encountered missense variant p.(Arg73Pro), often found in syndromic cases, was also associated with non-syndromic retinal degeneration. This study expands the current knowledge of *CFAP410*-associated ciliopathy by enriching its mutational landscape and supports its association with non-syndromic retinal degeneration.

## INTRODUCTION

The Cilia and Flagella Associated Protein 410 *(CFAP410)* gene (OMIM 603191), formerly known as *C21orf2*, is a ciliary gene of unclear specific function. Given its mapping position on chromosome 21, *CFAP410* was initially thought to play a role in the pathogenesis of several genetic diseases including Trisomy 21 (Down syndrome), but none of these associations have been confirmed^1–3^.

Functional genomic screens for ciliary gene identification^4,5^ combined with mutational screening in unsolved ciliopathy patients confirmed the essential role of CFAP410 in ciliogenesis. Individuals with bi-allelic pathogenic variants in this gene were reported to have Jeune syndrome^5^, a recessive skeletal ciliopathy (OMIM# 611263)^6,7^ also known as asphyxiating thoracic dystrophy. Affected individuals usually present with shortened ribs and a narrowed chest accompanied by other skeletal abnormalities but retinal degeneration and other nonskeletal features can be also present^7^.

Many ciliopathy cases harboring pathogenic *CFAP410* variants have been described to date. However, in 2015, Khan and colleagues described a specific phenotype of early-onset retinal dystrophy with macular staphyloma but without high myopia in three Saudi families with a history of consanguinity and carrying homozygous variants in *CFAP410*^8^. Since then, five other non-syndromic *CFAP410* cases have been reported as a consequence of mutational screens in large IRD cohorts ^9–11^*.* However, a conclusive association of *CFAP410* mutations with non-syndromic IRD has never been reached due to the small number of non-syndromic cases. In this study, we describe fourteen new families with early-onset non-syndromic retinal degeneration and two additional cases with a milder form of Jeune syndrome that confirm the phenotype expansion for bi-allelic variants in *CFAP410.* We also report eight novel variants in this gene, six of which are pathogenic or likely pathogenic.

## RESULTS

### Clinical phenotypes

**Fig. 1:**
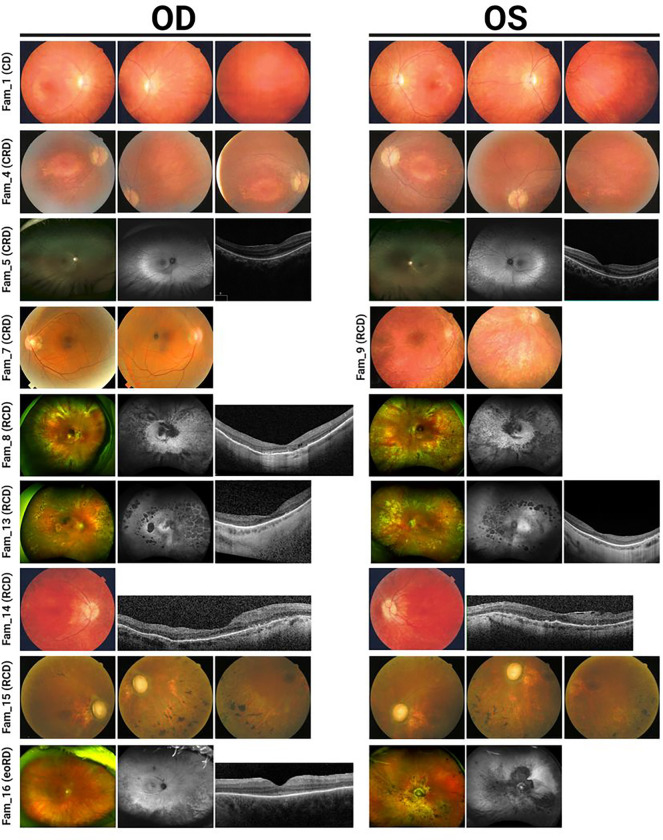
Clinical phenotypes of *CFAP410*-IRD patients. Images show fundus photos for a representative subset of individuals. Fundus autofluorescence and/or OCT imaging were available for five individuals (5, 8, 13, 14 and 16) and showed features consistent with the fundus findings and clinical diagnosis. The specific IRD phenotype of each patient is given in brackets (CD, cone dystrophy; CRD, cone-rod dystrophy; RCD, rod-cone dystrophy; eoRD, early-onset retinal dystrophy). Note the tapetal-like sheen in fundus images in proband 5 with CRD, and the morning glory disc in the left eye of proband 16 with eoRD.

Eight females and eight males with *CFAP410*-associated disease had clinical phenotypes falling into four diagnostic categories: Early-onset retinal dystrophy (eoRD; n=1), cone dystrophy (CD; n=1), cone-rod dystrophy (CRD; n=6), and rod-cone dystrophy (RCD; n=8) (see Fig. 1, Table 1, and Supplementary Table 1 for detailed clinical data). In most cases, the symptom onset occurred in childhood, prior to the age of 10, and at the initial clinical evaluation, the individuals were 9 to 71 years of age. The presenting symptom typically corresponded to the clinical diagnosis (for example, nyctalopia in RCD).

Visual acuity was significantly reduced at young ages regardless of clinical diagnosis. The youngest proband with CRD (proband 5) had visual acuity of 20/100 and 20/125 when evaluated at age 9, and the youngest proband with RCD (proband 9) had visual acuity of 20/100 in each eye at age 12. Except probands of families 10 and 11, no individual in the cohort had visual acuity better than 20/80 (see Supplementary Table 1), and fourteen eyes of eight individuals had visual acuity at or beyond the threshold of legal blindness at the initial evaluation.

When available, visual field data from Goldmann kinetic perimetry showed better overall preservation of visual fields in patients with clinical diagnoses of CD/CRD whereas most with RCD had constriction sparing only the central visual fields. Full-field ERGs were available for all patients. Individuals with clinical diagnoses of CD and CRD showed varying degrees of scotopic compromise with more severe photopic dysfunction; the scotopic responses for Proband 2 did show deterioration over two studies spanning 12 years. Individuals with RCD had severe generalized dysfunction of scotopic and photopic responses.

Fundus evaluation showed features that were typical for the retinal diagnosis (Fig. 1). Staphylomas were noted in two individuals (Probands 4, 8). Digital OCT images were available for eight individuals and showed significant attenuation and absence of photoreceptor bands particularly in the peripheral macula with relatively better preservation of foveal lamination. Visual acuity was lower than might be expected from the remaining structure with the structure vs. function dissociations in Probands 5, 13 (OS), and 16 particularly illustrative of this observation. OCT suggested posterior staphyloma in one individual for whom it was not noted on clinical exam (Proband 13).

Additional ophthalmic diagnoses included amblyopia (Proband 13), bilateral pseudophakia (Probands 11 and 12), history of strabismus surgery (Proband 14), and nystagmus (Probands 2, 16).

Skeletal abnormalities were present in two individuals: Proband 14 had thoracic skeletal abnormalities requiring surgical intervention, and proband 16 had bilateral hip dysplasia corrected with hip replacement. No other individuals had skeletal abnormalities present on imaging (Proband 3) or self-report. Proband 16 also had premature ovarian failure at age 30 as well as bilateral sensorineural hearing loss beginning in her 40s, but no other systemic diagnoses of note were present in the cohort.

### Rare *CFAP410* variants associated with non-syndromic early onset IRD

**Fig. 2:**
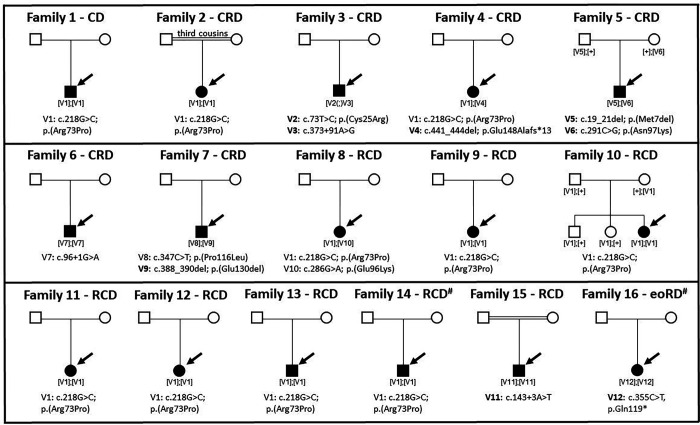
Pedigrees of the 14 *CFAP410* families described in this study. For each family (1–16), the specific IRD phenotype diagnosed is mentioned above each pedigree (CD, cone dystrophy; CRD, cone-rod dystrophy; RCD, rod-cone dystrophy; eoRD, early-onset retinal dystrophy). Mildly syndromic families 14 and 16 are indicated with a hashtag (#). Affected male and female subjects are represented with black squares or circles, respectively. Probands are indicated by a black arrow. Novel variants are indicated in bold. First cousin marriage is indicated by a double-line. All presented variants refer to the *CFAP410* transcript NM_004928.3. Biallelic inheritance was confirmed by familial segregation analysis (families 5 and 10), by ruling out deletion events in *CFAP410* bioinformatically (families 1, 2, 6, 9, 11, 12, 13, 14, 15, 16), by analysis of NGS pair-end reads (family 8), and by cloning and by using the gnomAD v2 Variant Co-Occurrence tool (families 4 and 7). In family 3 we could not confirm biallelic inheritance, thus variants are indicated as [V(;)V].

We identified 12 rare *CFAP410* variants (V1–12, MAF<0.0006) in 16 probands and their family members (see Fig. 2 and Table 2). No additional disease-causing variants were present in any of the currently known IRD genes ^12^ that were able to explain the clinical phenotype. The coding variants detected were truncating (p.Glu148Alafs*13 and p.Gln119*), missense (p.Arg73Pro, p.Glu96Lys, p.Asn97Lys, p.Pro116Leu, p.Cys25Arg), or leading to single amino acid deletions (p.Met7del and p.Glu130del), while the non-coding variants c.96+1G>A, c.143+3A>T and c.373+91A>G were located in *CFAP410* intron 2, 3 and 4, respectively.

Most of the detected variants were novel, except for p.Arg73Pro, c.96+1G>A, p.Glu96Lys, and p.Pro116Leu which were reported in the Leiden Open Variant Database (LOVD)^13^ in patients with syndromic and non syndromic IRD (See Supplementary Table 2 and 3). The p.Arg73Pro was the most commonly reported variant and also the most common in our cohort: present homozygously in 8 probands and heterozygously in two (families 4 and 8, see Fig. 1). However, this variant remains extremely rare in the general population, given the allele frequency in gnomAD v4 of 0.0005023^14^. Consanguinity was reported only in families 2 (c.218G>C, p.(Arg73Pro) ) and 15 (c.143+3A>T), in which the parents were third and first cousins, respectively. An additional proband 16 was homozygous for the c.355C>T, p.(Gln119*) variant., though no consanguinity was noted.

Biallelic inheritance in the homozygous cases was confirmed by familial segregation analysis (family 10) or by ruling out deletion events in *CFAP410* bioinformatically*.* Compound heterozygosity was confirmed by familial segregation analysis (family 5); analysis of NGS pair-end reads (family 8), and by cloning and by using the gnomAD v2 Variant Co-Occurrence tool (https://gnomad.broadinstitute.org/variant-cooccurrence) (families 4 and 7) (see Supplementary Fig. 1 and 2). Unfortunately, we could not use these methods to validate the phase of the variants identified in family 3, the c.73T>C; p.(Cys25Arg) and the c.373+91A>G. Both alleles were absent from gnomAD v2 and they were too far apart (~6 kb) to be cloned in one single fragment, given the limited quality of the historical DNA samples available. Only variant c.73T>C; p.(Cys25Arg) was present in one individual in the recently released version of GnomAD v4 (see Table 2), while variant c.373+91A>G was absent. However this data is too scarce to conclude definitively if these two variants are likely in *cis* or *trans*.

### Novel non-coding *CFAP410* variants lead to splicing defects

To investigate the effect of c.143+3A>T and c.373+91A>G on pre-mRNA splicing we generated wild-type and variant mini-gene splicing constructs, which were transfected into HEK293T cells. The effect on splicing was investigated by RT-PCR. Both variants were predicted to affect normal splicing according to multiple in silico tools, such as SpliceSiteFinder-like^15^, MaxEntScan^16^, NNSPLICE^17^, GeneSplicer^18^, Human Splicing Finder^19^, and SpliceAI^20^. Variant c.143+3A>T was predicted to disrupt the splice donor site of *CFAP410* exon 3, while c.373+91A>G was predicted to generate a strong splice acceptor site in intron 4 (see Supplementary Fig. 3 and 4).

**Fig. 3:**
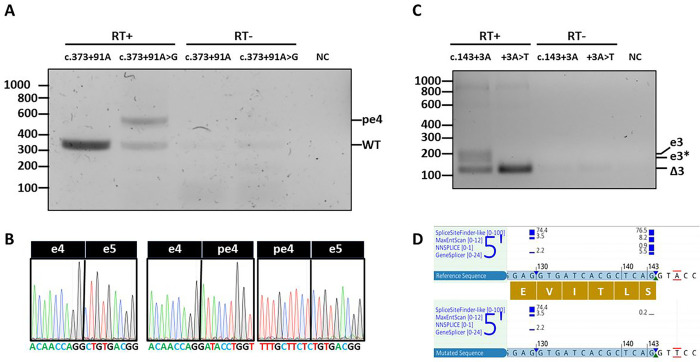
Functional validation of *CFAP410* splicing variants c.373+91A>G and c.143+3A>T. **(a)** RT-PCR showing the formation of a pseudoexon in intron 4 (pe4) in the construct containing the *CFAP410* c.373+91A>G variant compared to the wild-type (WT) band generated by the reference construct. **(b)** Sanger sequencing of the splice boundaries between exon 4 and 5, confirming the breakpoint of the pseudoexon. **(c)** RT-PCR showing the skipping of exon 3 (Δ3) in the construct containing the c.143+3A>T compared to the wild-type (WT) construct, which generates both a full and truncated version of exon 3, according to the splicing prediction **(d).**

The splicing assay confirmed the presence of aberrant splicing phenotypes for both variants (see Fig. 3). Indeed, exon 3 was skipped in the construct carrying the c.143+3A>T, while the splice acceptor created by c.373+91A>G resulted in the inclusion of a 200- base pair pseudoexon, previously predicted by SpliceAI (see Supplementary Fig. 4), in at least half of the transcripts according to our splicing assay (see Fig. 3A). Both splicing defects were classified as severe and fully penetrant, as they caused frameshift and premature stop codon in all generated transcripts (see Fig. 3).

### Protein modelling and prediction of missense variants

**Fig. 4:**
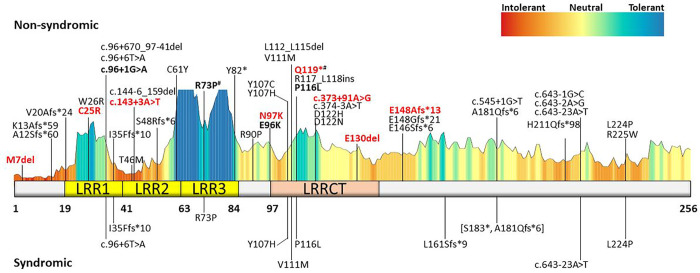
CFAP410 structure, mutation tolerance, and protein variants. CFAP410 secondary structure and distribution of known and novel disease variants found in affected individuals. Protein motifs and catalytic domain are highlighted using different colors, while variants were divided in two groups, depending whether they were found in syndromic or non-syndromic IRD patients. Known variants were retrieved from the Leiden Open Variation Database (LOVD) database. Variants reported in this study are in bold and novel variants are further highlighted in red. Variants p.S183* and p.A181Qfs*6, in square brackets, are part of the same complex allele as they result from the same nucleotide variant. # variants were found in mild syndromic cases. LRR, leucine-rich repeat; LRRCT, LRR C-terminal domain.

Variants in *CFAP410* have been associated with both syndromic (i.e., skeletal ciliopathies) and non-syndromic forms of retinal degeneration. To investigate whether this phenotypic variability was the consequence of a specific variant localization, we plotted the known 36 *CFAP410* variants reported in literature and the eight novel variants detected in our probands onto the secondary structure of the human CFAP410, a 256 amino acid protein (UniProtKB ID: O43822) (see Fig. 4 and Supplementary Table 3)^5,8–11,21–34^. Half of the analyzed 44 *CFAP410* variants were missense while the other half were either truncating or non-coding variants. The mutation tolerance at CFAP410 protein residues was analyzed using MetaDome (https://stuart.radboudumc.nl/metadome/)^35^, while the impact of specific missense variants on CFAP410 structure and function was predicted by tools like SIFT^36^, PolyPhen2^37^, CADD Phred^38^ REVEL^39^, and EVE^40^. In these analyses we did not detect mutational hotspots that would explain the observed phenotypic difference between syndromic and non-syndromic cases (see Fig. 4). Moreover, almost all the *CFAP410* variants detected in syndromic cases were also found in non-syndromic cases (see Fig. 4). The only exception was c.480_481insT, p.Leu161Serfs*9, a variant only detected in one family with severe skeletal abnormalities consistent with Jeune syndrome^5^. Consistent with prior reports, there was also not an apparent association in our cohort between the only recurrent variant, the p.(Arg73Pro) and the specific retinal phenotype, as this variant was associated with CD, CRD, and RCD.

Adopting the American College of Medical Genetics (ACMG) guidelines^41^, ten of the identified *CFAP410* variants were classified as pathogenic/likely pathogenic while p.Met7del and p.Asn97Lys were classified as variants of uncertain significance (VUSs) (Table 2).

## DISCUSSION

Here we describe sixteen probands with retinal degeneration associated with rare bi-allelic variants in *CFAP410,* a gene known for its association with recessive skeletal ciliopathies like Jeune Syndrome (JS) and Axial Spondylometaphyseal Dysplasia (SMDAX). Fourteen probands in our cohort did not have any syndromic features, and two individuals were recognized to have systemic findings related to *CFAP410* variants, noted only after genetic testing was performed. Of the 36 patients in the literature with *CFAP410*-associated retinopathy recently described and analyzed by Shinbashi et al, eleven lacked non-ocular features^42^. Our cases thus further support the association of variants in *CFAP410* with non-syndromic IRDs first described by Khan and colleagues^8^ and considerably expand the number of non-syndromic cases.

This report also expands upon prior reports of *CFAP410*-associated retinopathy. Patients in this cohort exhibited the spectrum of clinical diagnoses previously reported in the literature, with CRD and RCD equally represented. Both patient-reported symptoms and assessments of retinal function segregated into these different diagnostic categories and supported the differing ways in which CFAP410 dysfunction can manifest. A notable feature, regardless of clinical diagnosis, was the early disease onset, with symptoms beginning prior to the age of 10 years in those for whom a specific age could be recalled. Two thirds of the 36 patients described by Shinbashi et al. had symptom onset before age 18^42^. An additional aspect emphasized by the present cohort is the severity of central vision loss independent of clinical diagnosis: except probands of families 10 and 11, no other individuals in our cohort, including three between the ages of 9 and 13, had visual acuity better than 20/80 at the time of evaluation in our clinics. Indeed, the nystagmus observed in two patients, one with eoRD and one with CRD, is consistent with the early presence of central visual impairment. In the eight probands for whom spectral domain OCTs could be digitally reviewed, the degree of visual impairment was noted to be disproportionate to the degree of structural disruption. That is, although foveal structure was not normal in any of these patients, better visual acuity might have been anticipated. Posterior staphylomas disproportionate to the degree of myopia were present in three individuals as has previously been reported by Khan and others.

We identified eight novel variants, six of which are pathogenic/likely pathogenic, including the non-coding variant c.373+91A>G, which causes splicing defect and premature transcript truncation. Despite the spectrum of clinical variation, no genotype-phenotype correlations could be identified with regard to retinal phenotype.

The most recurrent variant in our cohort was the known p.Arg73Pro, found in eight homozygotes and two heterozygotes across clinical diagnoses. This variant is by far the most frequent change detected in *CFAP410* patients (see Supplementary Table 3) and it is the only described pathogenic variant localizing in the third leucine-reach repeat domain. Its total allele frequency is 0.0005023 in GnomAD v4, largely enriched in non-Finnish Europeans (772/810 alelles). The common origin for our cases carying this variant were Brittany and the British isles, particularly Ireland, suggesting a possible founder allele. The p.Arg73Pro variant is associated with a broad phenotypic spectrum (see Fig. 1). The proband from family 14, who was homozygous for the p.Arg73Pro variant had thoracic skeletal abnormalities for which two surgeries were required. Homozygosity for the p.Arg73Pro variant has also been reported previously in JS, SMDAX, and other syndromic IRD cases^5,23,33^. However, six additional probands in our cohort, homozygous for the p.Arg73Pro variant lacked extraocular features.

Proband from family 16, homozygous for the p.Gln119* change, suffered from bilateral hip dysplasia, asymmetric bilateral hearing loss, and early ovarian failure. The p.Gln119* change introduces a stop codon in exon 4, of the 7 exon *CFAP410* gene, which most likely leads to nonsense-mediated decay (NMD)^43–47^ of the whole transcript and thus is considered a null allele. Since proband from family 16 does not have any functional CFAP410 protein, we consider her overall phenotype to be relatively mild compared to Jeune syndrome cases^6,7^. The other truncating variant detected in this study, p.Glu148Alafs*13, is located in exon 5 and is also thought to lead to transcript degradation through NMD and thus a null allele. This variant appeared in *trans* with the p.Arg73Pro change in the non-syndromic proband of family 4. Such genotypes were also reported in the past to lead to more severe phenotypes^5,23,25,32,33^. The two non-coding variants validated in our study, c.143+3A>T and c.373+91A>G, showed a full and partial splicing defect on a mini-gene splicing assay, respectively. Both cases presented with a non-syndromic retinal degeneration (see Fig.1 and Supplementary Fig. 5). It is important to mention that under the experimental settings of our splicing assay, namely testing the effect of a variant in a limited genomic context, the strength of the observed splicing effect is approximate and we cannot rule out that the c.143+3A>T might have a less severe molecular effect when tested in a larger genomic context.

Since the actual function of CFAP410 remains unknown, it is still unclear what are the molecular mechanisms able to explain the phenotypic heterogeneity observed in patients carrying mutations in this gene. It has been hypothesized that this variability might be the consequence of the functional interaction of CFAP410 with two other proteins NEK1 and SPATA7, as they form a protein complex localized to photoreceptor ciliary structures in multiple species including humans^5,8,10^. Therefore, it seems likely that this protein complex might have different targets, some of which tissue-specific, eventually resulting in different clinical signs^5,33,48^.The simple comparison of *CFAP410* variant distribution between syndromic and non-syndromic IRD cases has proven inconclusive. Moreover, the small size of our pedigrees did not allow us to observe phenotypic differences among affected members of the same family. We cannot explain the reason behind a milder than expected systemic phenotype of cases carrying homozygous p.Arg73Pro variants or null alleles. We hypothesize that another protein may be able to partially substitute for the CFAP410 function, which can be facilitated by modifying variants in the gene coding for that protein.

In conclusion, our data validate the phenotypic expansion caused by pathogenic variants in *CFAP410* and expand the mutation landscape of this gene by providing novel coding and non-coding variants in this ciliopathy gene.

## METHODS

### Ethics statement

The study was approved by the institutional review board of all participating institutions (Committees of Protection of Persons Ile de France V for families 6, 10, 11, and 12, and Partners HealthCare System for all remaining families) and adhered to the Declaration of Helsinki. Informed consent was obtained from all individuals on whom genetic testing and further molecular evaluations were performed.

### Clinical evaluation

Sixteen probands with autosomal recessive retinal degeneration were enrolled in this study. Twelve probands were ascertained from Massachusetts Eye and Ear, and another four from the National Reference Centre of Rare Diseases at Quinze-Vingts National Hospital. Clinical evaluation was performed by experienced ophthalmologists according to previously published protocols and included functional and structural assessments^49–52^.

### Genetic analysis

All probands analyzed in this study, except the ones of families 6, 10, 11, and 12, are part of a historical cohort that underwent clinical evaluation in the Inherited Retinal Disorder Service at (MEE; Boston, MA) in the 1990s and early 2000s. Blood samples were obtained from probands, and when possible, their parents. DNA was isolated from peripheral blood lymphocytes by standard procedures. Probands of four families (5, 9, 13, 15) were sequenced using the Genetic Eye Disease (GEDi) panel, described previously^53,54^. The GEDi version used in this study (v6) targeted exons of 327 known and candidate IRD genes (see Supplementary Table 4)^55^. The NGS data from the GEDi panel was analyzed using Genome Analysis Toolkit (GATK) version 3 ^56^ and annotated using the Variant Effect Predictor (VEP) tool ^57^ with additional annotations taken from the Genome Aggregation Database (gnomAD)^14^, the Genomic Evolutionary Rate Profiling (GERP)^58^, SIFT^36^, PolyPhen2^37^, CADD Phred^38^ and retinal expression^59^. To detect possible copy number variations, gCNV software was used as before^60^. Relatedness of the families sequenced with GEDi panel was excluded using Peddy^61^. Exome sequencing (ES) for eight probands was performed at the Center for Mendelian Genomics at the Broad Institute of the Massachusetts Institute of Technology and Harvard using methodology described previously ^54^. WES data were aligned to hg38 and variants were called using the GATK HaplotypeCaller package version 3.5 (https://software.broadinstitute.org/gatk/). Data were displayed and analyzed with an online tool (https://seqr.broadinstitute.org).

Probands from families 6, 10, 11, and 12 had been screened applying a customized NGS panel as reported before^62^ updated regularly to include newly IRD-associated genes.

### Variant validation and phasing

All presented variants refer to the *CFAP410* transcript NM_004928.3. Variant segregation was performed by Sanger sequencing (see Supplementary Table 5) or analysis of NGS reads. Although the variants detected in probands of families 4 and 7 were considered in *trans* according to the gnomAD browser Variant Co-Occurrence tool (https://gnomad.broadinstitute.org/variant-cooccurrence), they were further phased by cloning and Sanger sequencing. Briefly, genomic DNA from the proband was amplified using Phusion (New England Biolabs) and primers spanning the region containing all variants. The amplified fragment was then cloned into the pCR2.1 plasmid, TA cloning kit (Invitrogen) and Sanger sequenced. Sanger sequencing was performed on ABI 3730xl (Applied Biosystems) using BigDye Terminator v3.1 kits (Life Technologies). Sequence analysis was done using SeqManPro (Lasergene 11, DNAStar Madison, WI, USA), in which variants were considered to be *in trans* when they were not present on the same clone.

### Protein modelling, prediction of missense variants, and variant classification.

The mutation tolerance at CFAP410 protein residues was analyzed using MetaDome (https://stuart.radboudumc.nl/metadome/)^35^, while the impact of specific missense variants on CFAP410 structure and function, was predicted by using five prediction algorithms: SIFT^36^, PolyPhen2^37^, CADD Phred^38^ REVEL^39^, and EVE^40^. Variants were finally classified according to the (ACMG) guidelines^41^.

## Data Availability

Variants are available through ClinVar (Accession code).
